# ﻿Three new species of the genus *Trilacuna* Tong & Li, 2007 (Araneae, Oonopidae) from Yunnan Province, China

**DOI:** 10.3897/zookeys.1174.106130

**Published:** 2023-08-14

**Authors:** Jimeng Ma, Dongju Bian, Yanfeng Tong, Zizhong Yang, Zhisheng Zhang

**Affiliations:** 1 Life Science College, Shenyang Normal University, Shenyang 110034, Liaoning, China Shenyang Normal University Shenyang China; 2 Key Laboratory of Forest Ecology and Management, Institute of Applied Ecology, Chinese Academy of Sciences, Shenyang 110016, China Key Laboratory of Forest Ecology and Management, Institute of Applied Ecology, Chinese Academy of Sciences Shenyang China; 3 National-Local Joint Engineering Research Center of Entomoceutics, Dali University, Yunnan Dali, 671000, China Dali University Dali China; 4 Key Laboratory of Eco-environments in Three Gorges Reservoir Region (Ministry of Education), School of Life Sciences, Southwest University, Chongqing 400715, China Southwest University Chongqing China

**Keywords:** Asia, distribution, goblin spiders, morphology, taxonomy

## Abstract

Three new species of the genus *Trilacuna* Tong & Li, 2007, *T.cangshan* Tong, Yang & Zhang, **sp. nov.** (♂), *T.wumanshan* Tong, Yang & Zhang, **sp. nov.** (♂), and *T.xiaoheishan* Tong, Yang & Zhang, **sp. nov.** (♂♀) are described from Yunnan, China. Descriptions, diagnoses, and photographs are provided.

## ﻿Introduction

The family Oonopidae Simon, 1890 is composed of tiny spiders between 1.0 and 3.0 mm. They have a nearly worldwide distribution, occurring mainly in leaf litter, under bark, and in the tree canopy (Jocqué and Dippenaar-Schoeman 2006; Ubick and Dupérré 2017). Oonopidae is among the nine most diverse spider families with 1891 extant described species in 115 genera (WSC 2023).

The genus *Trilacuna* Tong & Li, 2007 currently comprises 39 species. All species are known from Bhutan, China, India, Indonesia (Sumatra), Iran, Korea, Malaysia, Myanmar, Nepal, Pakistan, Thailand, and Vietnam (Tong and Li 2007; Eichenberger and Kranz-Baltensperger 2011; Grismado et al. 2014; Malek-Hosseini et al. 2015). In China, the genus is represented by 17 species, of which seven species are from Chongqing, one species from Guizhou, and nine species from Yunnan Province (Tong et al. 2019; Huang et al. 2020, 2021; Wang et al. 2021). In this paper, three new *Trilacuna* species collected from Yunnan Province are described and illustrated.

## ﻿Materials and methods

The specimens were examined using a Leica M205C stereomicroscope. Details were studied under an Olympus BX51 compound microscope. Photos were taken with a Canon EOS 550D zoom digital camera (18 megapixels) mounted on an Olympus BX51 compound microscope. Vulvae were cleared in lactic acid. Scanning electron microscope images (SEM) were taken under high vacuum with a Hitachi S-4800 after critical point drying and gold-palladium coating. All measurements were taken using an Olympus BX51 compound microscope and are in millimeters. Taxonomic descriptions follow Tong et al. (2020). Type materials are deposited in Shenyang Normal University (SYNU) in Shenyang, China.

The following abbreviations are used in the text and figures: **ALE** = anterior lateral eyes; **ap** = apodemes; **as** = anterior sclerite; **blp** = basal leaf-shaped projection; **brc** = branch with row of combs; **bsh** = basal short “hairs”; **bth** = basal thin “hairs”; **cdb**= slightly curved distal branch; **db** = dorsal branch; **dth** = distal thick “hairs”; **ehb** = elevated hair base; **glo** = globular structure; **lb** = lateral branch; **lcb** = lateral curved branch; **ldi** = labium deep incision; **ldp** = large dorsal prong; **lh** = lateral “hairs”; **lmb** = long median branch; **mb** = median branch; **PME** = posterior median eye; **psp** = posterior spiracle; **sar** = sclerotized, recurved arches; **sdb** = short dorsal branch; **tmb** = thin median branch; **tsc** = transverse sclerite; **vbl** = ventral broad lobes; **vp** = ventral projection; **wfb** = distally widened flat branch.

## ﻿Taxonomy

**Family Oonopidae Simon, 1890**

### ﻿Genus Trilacuna

Taxon classificationAnimaliaAraneaeOonopidae

Tong & Li, 2007

6221AEA4-5CAE-5DB6-AEF9-0179C3D8F9F5

#### Type species.

*Trilacunarastrum* Tong & Li, 2007 from Yunnan, China.

#### Diagnosis.

See Tong et al. (2020).

#### Composition.

42 species, including three described here.

#### Distribution.

Iran to the Korean Peninsula and south to Sumatra.

### ﻿ Trilacuna cangshan

Taxon classificationAnimaliaAraneaeOonopidae

Tong, Yang & Zhang sp. nov.

F53E1A6F-F6C2-55BB-B628-BC6FD1C4E268

https://zoobank.org/D00C8223-91F4-4A42-8F73-AAAE7412F090

Figs 1 , 2 , 8

#### Type material.

***Holotype*** ♂ (SYNU-660): China, Yunnan Province, Dali Bai Autonomous Prefecture, Dali City, Cangshan Mountain, post-fire forest in 1999, 25°38′30″N, 100°08′04″E, Z. Yang leg., 2/11/2009.

#### Diagnosis.

**Figure 1.**
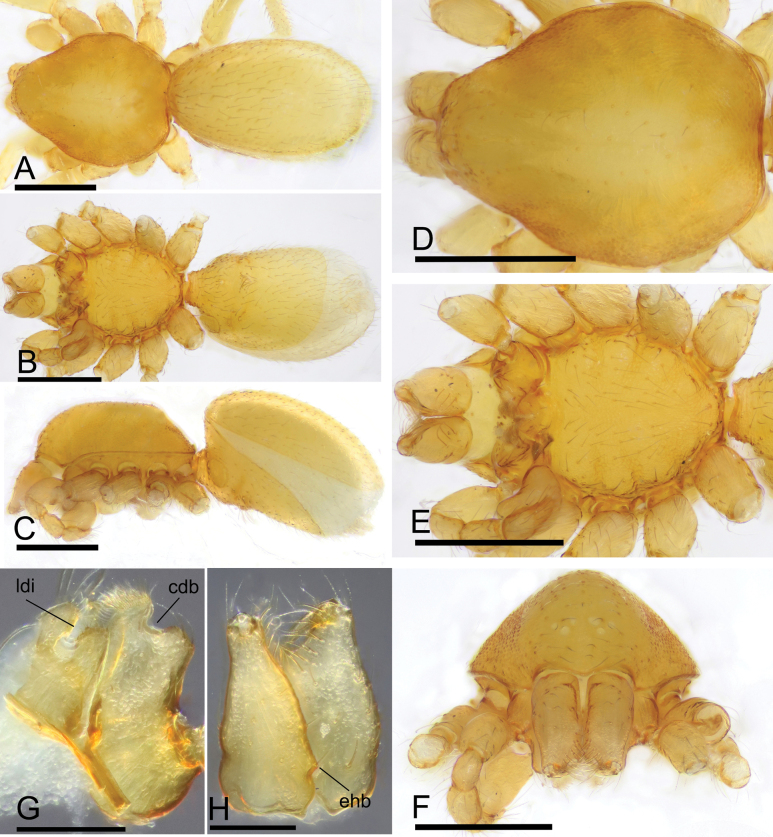
*Trilacunacangshan* sp. nov., male holotype **A–C** habitus in dorsal, ventral, and lateral views **D–F** prosoma in dorsal, ventral, and anterior views **G** labium and endites in ventral view **H** chelicerae, slightly oblique lateral view. Abbreviations: cdb = slightly curved distal branch; ehb = elevated hair base; ldi = labium deep incision. Scale bars: 0.4 mm (**A–F**); 0.2 mm (**G, H**).

Males of the new species are similar to those of *T.bawan* Tong, Zhang & Li, 2019 in the shape of palp, but they can be distinguished by the reduced eyes (Fig. 1D, F) vs normal, the smooth sternum (Fig. 1E) vs with grooves at posterior part, and the slightly elevated epigastric region (Fig. 1B, C) vs epigastric region strongly elevated (Tong et al. 2019: fig. 1A, E, G–I).

#### Description.

**Figure 2.**
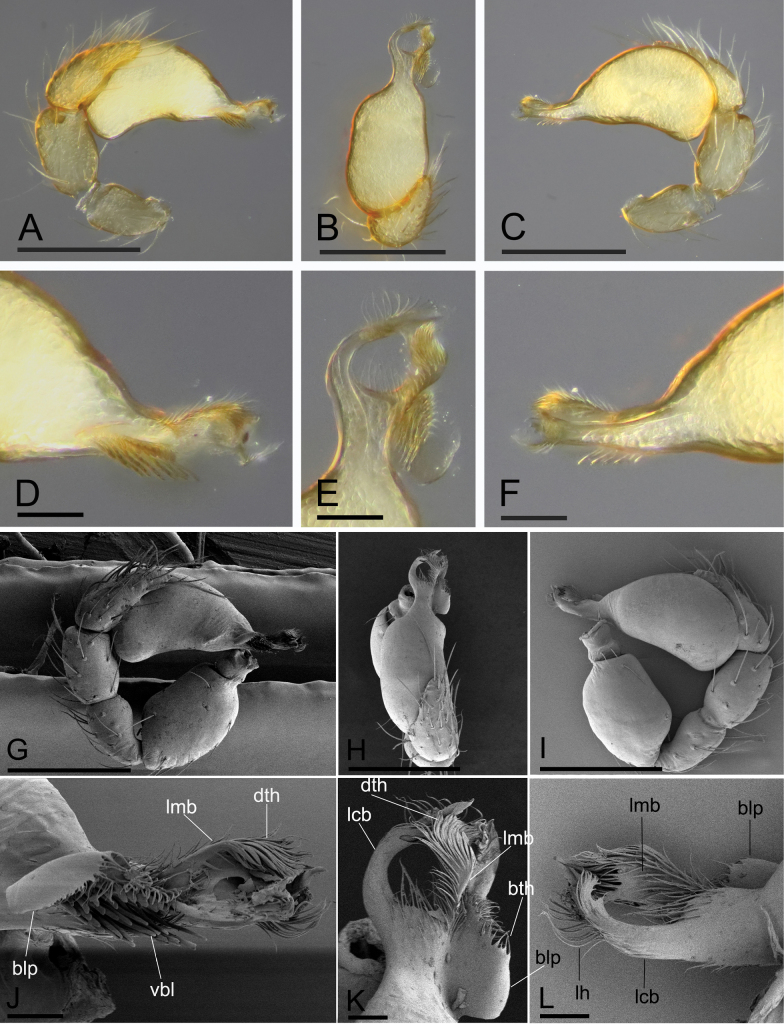
*Trilacunacangshan* sp. nov., left male palp **A–F** (light) and right male palp **G–L** (SEM, images flipped horizontally) **A, G** prolateral view **B, H** dorsal view **C, I** retrolateral view **D, J** distal part of bulb, prolateral view **E, K** distal part of bulb, dorsal view **F, L** distal part of bulb, retrolateral view. Abbreviations: blp = basal leaf-shaped projection; bth = basal thin “hairs”; dth = distal thick “hairs”; lcb = lateral curved branch; lh = lateral “hairs”; lmb = long median branch; vbl = ventral broad lobes. Scale bars: 0.2 mm (**A–C, G–I**); 0.02 mm (**D–F, J–L**).

**Male (holotype). *Body***: yellow, legs lighter; habitus as in Fig. 1A–C; body length 1.76. ***Carapace***: 0.78 long, 0.67 wide; sides granulate, lateral margin rebordered (Fig. 1D, F). ***Eyes***: vestigial, only visible in frontal view (Fig. 1D, F). ***Mouthparts***: chelicerae straight, proximal region with one seta with elevated base; labium rectangular, anterior margin deeply incised; endites slender, distally branched (Fig. 1E, G, H). ***Sternum***: surface finely reticulated (Fig. 1E). ***Abdomen***: 1.01 long, 0.60 wide; booklung covers ovoid, surface smooth; dorsal scutum not fused to epigastric scutum; sperm pore situated at level of anterior spiracles; apodemes present, posterior spiracles connected by groove; epigastric region slightly elevated, with a cluster of densely short setae (Fig. 1A–C). ***Palp***: orange; 0.64 long (0.20, 0.13, 0.13, 0.18); femur greatly elongated (width/length = 0.64); bulb kidney-shaped, tapering apically; psembolus with basal leaf-shaped projection (blp) and ventral broad lobes (vbl); with dorsal median branch (lmb) and retrolateral, long curved branch (lcb) (Fig. 2).

**Female.** Unknown.

#### Etymology.

The specific name is a noun in apposition taken from the type locality.

#### Distribution.

Known only from the type locality, Yunnan Province, China (Fig. 8).

### ﻿ Trilacuna wumanshan

Taxon classificationAnimaliaAraneaeOonopidae

Tong, Yang & Zhang sp. nov.

EB818C04-C300-54F5-A09A-94486B304ED5

https://zoobank.org/05309BE8-2203-4DD0-86B3-88B00D9CFC05

Figs 3 , 4 , 8

#### Type material.

***Holotype*** ♂ (SYNU-661): China, Yunnan Province, Lincang City, Cangyuan Wa Autonomous County, Banhong township, Wuman Hill, 23°15′15″N, 99°05′52″E, Z. Yang leg., 13/5/2021.

#### Diagnosis.

**Figure 3.**
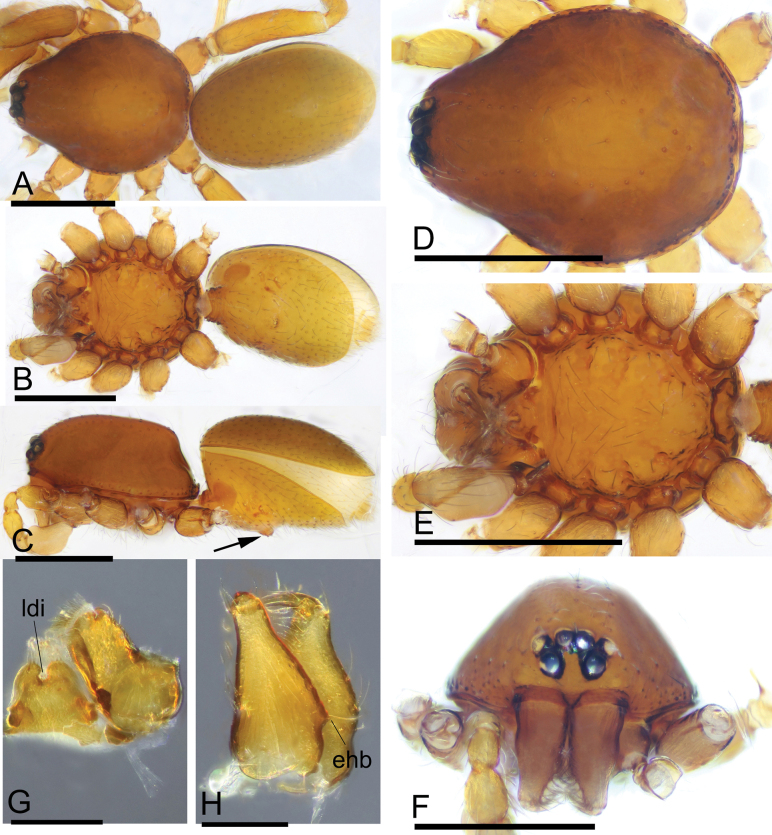
*Trilacunawumanshan* sp. nov., male holotype **A–C** habitus in dorsal, ventral, and lateral views, arrow shows the elevated epigastric region **D–F** prosoma in dorsal, ventral, and anterior views **G** labium and endites in ventral view **H** chelicerae in slightly oblique lateral view. Abbreviations: ehb = elevated hair base; ldi = labium deep incision. Scale bars: 0.4 mm (**A–F**); 0.2 mm (**G, H**).

**Figure 4.**
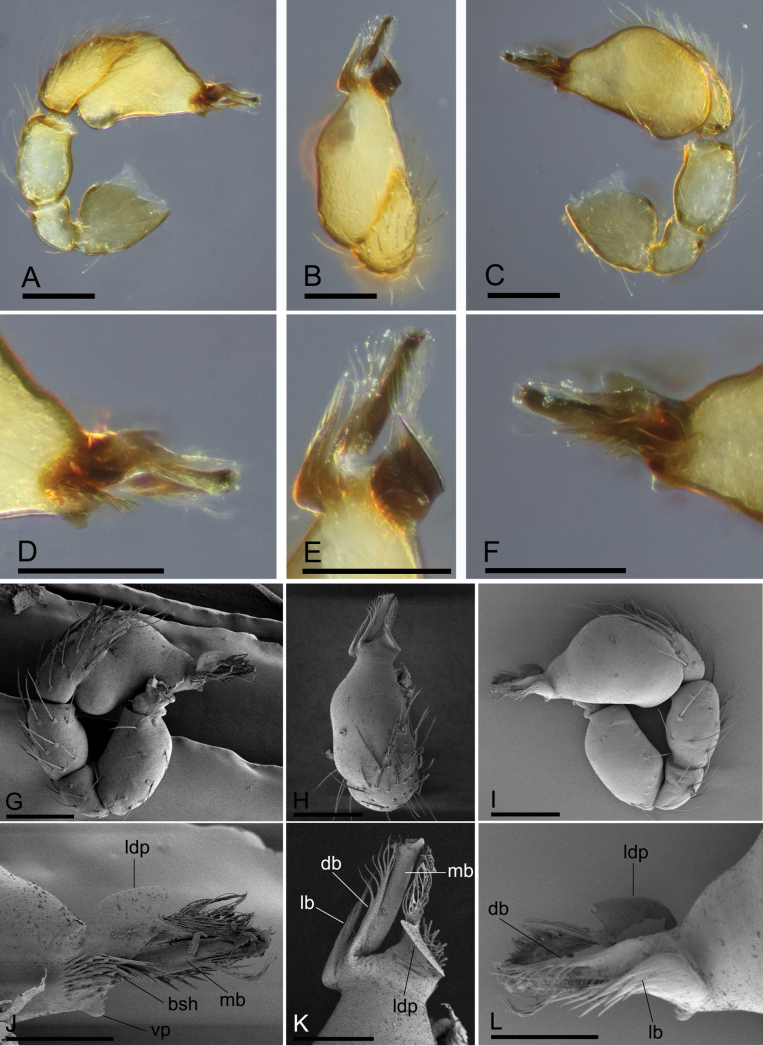
*Trilacunawumanshan* sp. nov., left male palp, **A–F** (light) and **G–L** (SEM) **A, G** prolateral view **B, H** dorsal view **C, I** retrolateral view **D, J** distal part of bulb, prolateral view **E, K** distal part of bulb, dorsal view **F, L** distal part of bulb, retrolateral view. Abbreviations: bsh = basal short “hairs”; db = dorsal branch; lb = lateral branch; ldp = large dorsal prong; mb = median branch; vp = ventral projection. Scale bars: 0.1 mm (**A–C, G–I**); 0.05 mm (**D–F, J–L**).

The new species is similar to *T.longtankou* Tong & Li, 2020 in the shape of the male palp, but it can be distinguished by the smooth sternum (Fig. 3B) vs sternum with two rows of paddle-shaped setae, and the large dorsal prong and the narrow lateral and dorsal branches of psembolus (Fig. 4J, K) vs a basal branch and several broad branches of psembolus (Huang et al. 2020: fig. 3E, F, H).

#### Description.

**Male (holotype). *Body***: yellow-brown, chelicerae and sternum lighter, legs yellow; habitus as in Fig. 3A–C; body length 1.44. ***Carapace***: 0.70 long, 0.56 wide; sides smooth, lateral margin rebordered (Fig. 3D). ***Eyes***: ALE largest, PME smallest; ALE separated from edge of carapace by 1.0 diameters (Fig. 3D, F). ***Mouthparts***: chelicerae straight, proximal region with one hair with elevated hair base; labium rectangular, anterior margin deeply incised; endites slender, distally not branched (Fig. 3E, G, H). ***Sternum***: surface finely smooth (Fig. 3E). ***Abdomen***: 0.72 long, 0.47 wide; booklung covers ovoid, surface smooth; dorsal scutum not fused to epigastric scutum; sperm pore situated at level of posterior spiracles; apodemes present, posterior spiracles not connected by groove; epigastric region slightly elevated (Fig. 3A–C). ***Palp***: orange; 0.54 long (0.16, 0.09, 0.12, 0.17); femur greatly elongated (width/length = 0.60); bulb triangle, tapering anteriorly; psembolus with large dorsal prong (ldp), a cluster of basal short “hairs” (bsh), and a small ventral projection (vp); with a broad median branch (mb), a narrow lateral branch (lb) and a dorsal branch (db) (Fig. 4).

**Female.** Unknown.

#### Etymology.

The specific name is a noun in apposition taken from the type locality.

#### Distribution.

Known only from the type locality, Yunnan Province, China (Fig. 8).

### ﻿ Trilacuna xiaoheishan

Taxon classificationAnimaliaAraneaeOonopidae

Tong, Yang & Zhang sp. nov.

5EFF157C-5FA6-58C5-8E34-8A806816FA0D

https://zoobank.org/F599EE3B-6643-418A-8967-132D41CD1D3B

Figs 5 , 6 , 7 , 8

#### Type material.

***Holotype*** ♂ (SYNU-653): China, Yunnan Province, Baoshan City, Longling County, Xiaoheishan Natural Reserve, Z. Li & L. Wang leg., 17/2/2011; ***Paratypes*** 1 ♂ (SYNU-654),1 ♀ (SYNU-655), 2 ♀ (SYNU-656-657), same data as holotype.

#### Diagnosis.

**Figure 5.**
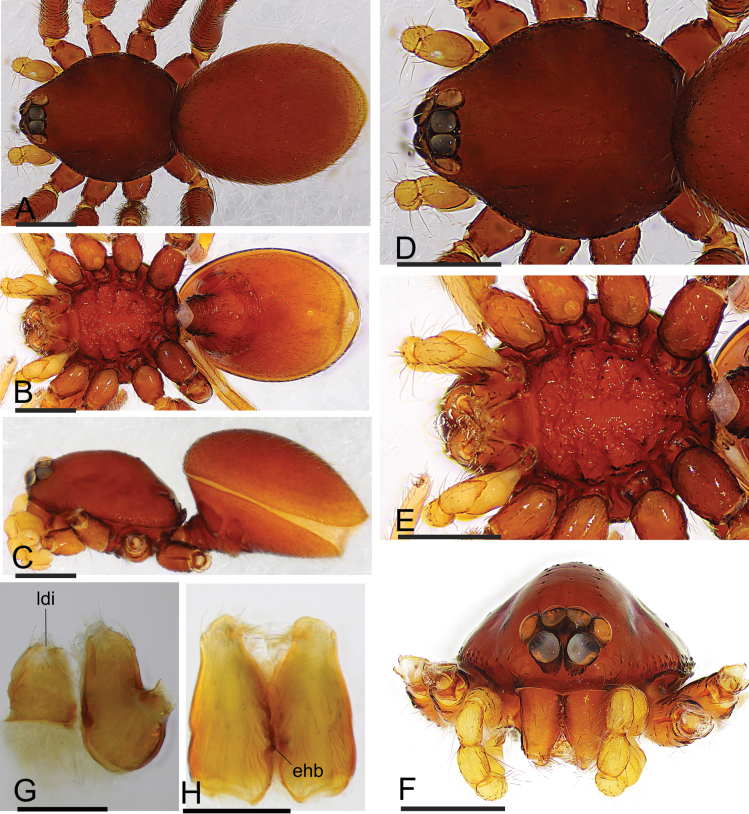
*Trilacunaxiaoheishan* sp. nov., male holotype **A–C** habitus in dorsal, ventral, and lateral views **D–F** prosoma in dorsal, ventral, and anterior views **G** labium and endites in ventral view **H** chelicerae, anterior view. Abbreviations: ehb = elevated hair base; ldi = labium deep incision. Scale bars: 0.4 mm (**A–F**); 0.2 mm (**G, H**).

**Figure 6.**
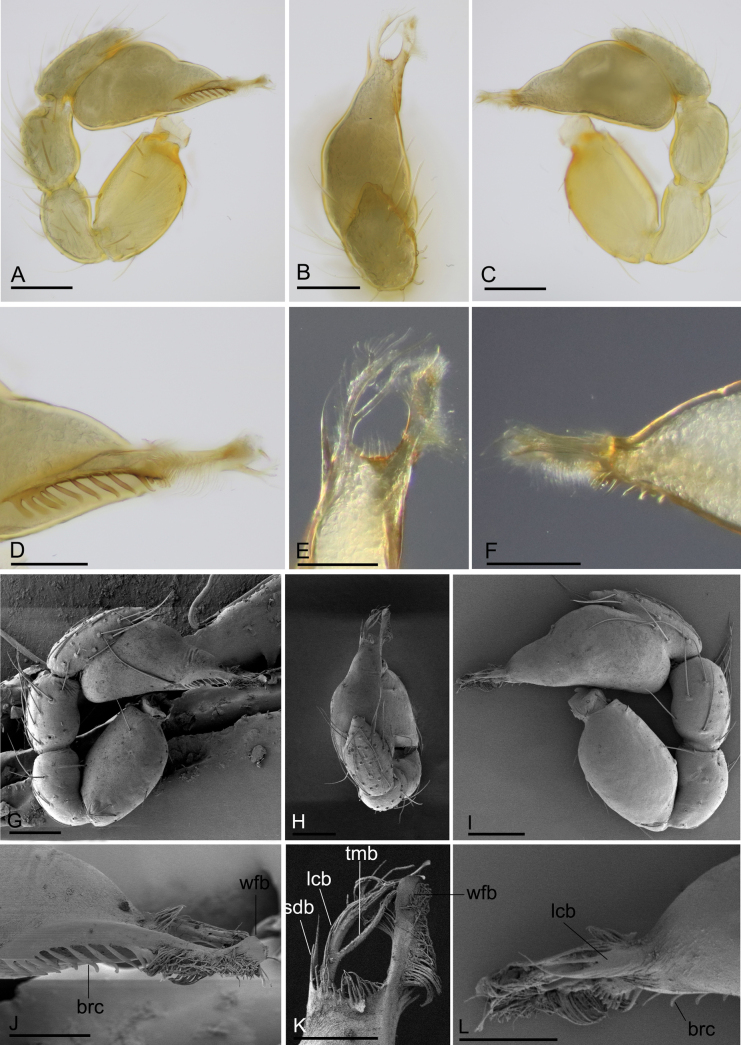
*Trilacunaxiaoheishan* sp. nov., left male palp **A–F** (light) and **G–L** (SEM) **A, G** prolateral view **B, H** dorsal view **C, I** retrolateral view **D, J** distal part of bulb, prolateral view **E, K** distal part of bulb, dorsal view **F, L** distal part of bulb, retrolateral view. Abbreviations: brc = branch with row of combs; sdb = short dorsal branch; lcb = lateral curved branch; tmb = thin median branch; wfb = distally widened flat branch. Scale bars: 0.1 mm (**A–C, G–I**); 0.05 mm (**D–F, J–L**).

**Figure 7.**
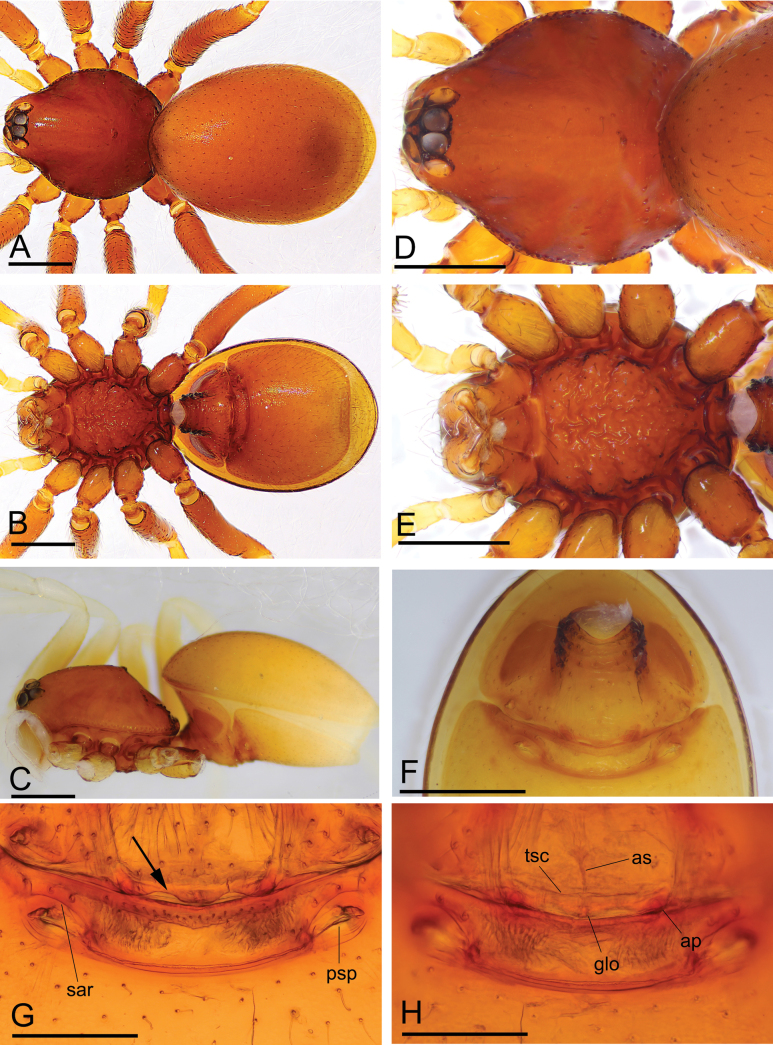
*Trilacunaxiaoheishan* sp. nov., female paratype **A–C** habitus in dorsal, ventral, and lateral views **D, E** prosoma in dorsal and ventral views **F** abdomen in ventral view **G, H** copulatory organ in ventral and dorsal views, arrow shows the curved posterior margin. Abbreviations: ap = apodemes; as = anterior sclerite; glo = globular structure; psp = posterior spiracle; sar = sclerotized, recurved arches; tsc = transverse sclerite. Scale bars: 0.4 mm (**A–F**); 0.2 mm (**G, H**).

Males of the new species are similar to those of *T.werni* Eichenberger, 2011 in the shape of the male palp, but they can be distinguished by the branch of psembolus with row of combs (Fig. 6D, J) vs a groove with row of lobes (Eichenberger and Kranz-Baltensperger 2011: fig. 12A, B, E, I), and the unfused abdominal dorsal and ventral scuta (Fig. 5C) vs dorsal scutum fused to the epigastric scutum (Eichenberger and Kranz-Baltensperger 2011: fig. 11C). Females can be distinguished from the other *Trilacuna* species by the curved posterior margin of epigastric scutum (Fig. 7F, G) vs roundly bent.

#### Description.

**Male (holotype). *Body***: reddish-brown, chelicerae and sternum lighter, legs yellow; habitus as in Fig. 5A–C; body length 2.37. ***Carapace***: 1.08 long, 0.89 wide; sides smooth, lateral margin rebordered (Fig. 5D). ***Eyes***: ALE largest, PME smallest; ALE separated from edge of carapace by 0.93 diameters (Fig. 5D, F). ***Mouthparts***: chelicerae straight, proximal region with one hair with elevated hair base; labium rectangular, anterior margin deeply incised; endites slender, distally not branched (Fig. 5E, G, H). ***Sternum***: surface strongly rugose (Fig. 5E). ***Abdomen***: 1.36 long, 0.92 wide; booklung covers ovoid, surface smooth; apodemes present; sperm pore situated at level of anterior spiracles; posterior spiracles connected by groove (Fig. 5A–C). ***Palp***: orange; 0.79 long (0.24, 0.15, 0.17, 0.23); femur greatly elongated (width/length = 0.61); bulb triangle, tapering anteriorly; psembolus with a long distally widened flat branch (wfb), with a row of combs (brc) at basal half of the branch and numerous “hairs” on distal half of the branch; with a retrolateraly long curved branch (lcb), a short dorsal branch (sdb) and a thin median branch (tmb) (Fig. 6).

**Female (paratype, SYNU-655). *Body***: habitus as in Fig. 7A–C; slightly larger than male; body length 2.57. ***Carapace***: 1.09 long, 0.91 wide. ***Mouthparts***: endites unmodified. ***Abdomen***: 1.60 long, 1.13 wide. ***Epigastric area***: middle part of posterior margin of epigastric scutum curved, with sclerotized recurved arches (sar) between posterior spiracles (psp) (Fig. 7F, G). ***Endogyne***: with narrow, transversally elongated sclerite (tsc); anterior T-shaped sclerite (as) and posterior small globular structure (glo) (Fig. 7G, H).

#### Etymology.

The specific name is a noun in apposition taken from the type locality.

#### Distribution.

**Figure 8.**
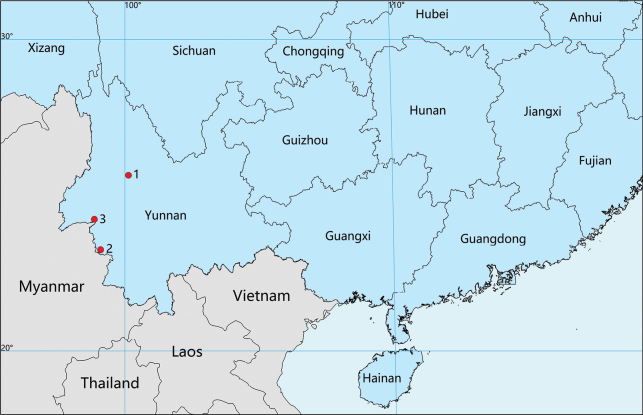
Distribution records of three new species from Yunnan, China. 1. *Trilacunacangshan* sp. nov.; 2. *Trilacunawumanshan* sp. nov.; 3. *Trilacunaxiaoheishan* sp. nov.

Known only from the type locality, Yunnan Province, China (Fig. 8).

## Supplementary Material

XML Treatment for Trilacuna

XML Treatment for Trilacuna cangshan

XML Treatment for Trilacuna wumanshan

XML Treatment for Trilacuna xiaoheishan

## ﻿Additional information

### Author ORCIDs

Jimeng Ma https://orcid.org/0009-0008-4579-3506

Dongju Bian https://orcid.org/0000-0002-9324-8026

Yanfeng Tong https://orcid.org/0000-0002-4348-7029

Zizhong Yang https://orcid.org/0000-0002-2143-8226

Zhisheng Zhang https://orcid.org/0000-0002-9304-1789

### Data availability

All of the data that support the findings of this study are available in the main text.
